# Muscle Releases Alpha-Sarcoglycan Positive Extracellular Vesicles Carrying miRNAs in the Bloodstream

**DOI:** 10.1371/journal.pone.0125094

**Published:** 2015-05-08

**Authors:** Michele Guescini, Barbara Canonico, Francesco Lucertini, Serena Maggio, Giosué Annibalini, Elena Barbieri, Francesca Luchetti, Stefano Papa, Vilberto Stocchi

**Affiliations:** 1 Department of Biomolecular Sciences, University of Urbino *Carlo Bo*, Urbino, Italy; 2 Department of Human, Environment and Nature Science, University of Urbino *Carlo Bo*, Urbino, Italy; IRCCS-Policlinico San Donato, ITALY

## Abstract

In the past few years, skeletal muscle has emerged as an important secretory organ producing soluble factors, called myokines, that exert either autocrine, paracrine or endocrine effects. Moreover, recent studies have shown that muscle releases microRNAs into the bloodstream in response to physical exercise. These microRNAs affect target cells, such as hormones and cytokines. The mechanisms underlying microRNA secretion are poorly characterized at present. Here, we investigated whether muscle tissue releases extracellular vesicles (EVs), which carry microRNAs in the bloodstream under physiological conditions such as physical exercise. Using density gradient separation of plasma from sedentary and physically fit young men we found EVs positive for TSG101 and alpha-sarcoglycan (SGCA), and enriched for miR-206. Cytometric analysis showed that the SGCA+ EVs account for 1–5% of the total and that 60–65% of these EVs were also positive for the exosomal marker CD81. Furthermore, the SGCA-immuno captured sub-population of EVs exhibited higher levels of the miR-206/miR16 ratio compared to total plasma EVs. Finally, a significant positive correlation was found between the aerobic fitness and muscle-specific miRNAs and EV miR-133b and -181a-5p were significantly up-regulated after acute exercise. Thus, our study proposes EVs as a novel means of muscle communication potentially involved in muscle remodeling and homeostasis.

## Introduction

It is a long held belief that during physical activity contracting skeletal muscle communicates with other organs *via* humoral factors in order to meet the increased energy demand. Indeed, researchers have been searching for contraction-induced factors for more than fifty years [1]. However, it was only in the past decade that a pioneer study by Pedersen *et al*. [2] demonstrated that the cytokine IL-6 is produced and released by muscle fibers in response to acute aerobic exercise. Subsequent studies have shown that, in addition to IL-6, contracting muscle releases other cytokines and several hundred proteins [3,4]. These muscle-derived factors have the capacity to exert endocrine/paracrine effects; hence, they are now recognized as myokines [5].

Skeletal muscle is the most important tissue in the human body in terms of total weight; it constitutes approximately 40% of body weight in lean men and women. Therefore, the discovery that contracting muscle is a myokine-producing organ has opened up a whole new field of research on skeletal muscle [6]. Indeed, recent studies have reported that muscle may also release microRNAs (miRNAs) into the bloodstream in response to physical exercise. Baggish *et al*. [7] reported the induction of unique signatures of circulating miRNAs in response to a single bout of aerobic exercise and after aerobic exercise training.

MiRNAs function as important regulators of a wide range of cellular processes by modulating gene expression at transcriptional or post-transcriptional level by binding the 3’-untranslated regions of mRNA transcripts and thereby repressing mRNA translation and/or stimulating mRNA degradation [8].

From screening for new miRNAs came the realization that some miRNAs have a restricted pattern of expression, among these miR-1, -133a, -133b, -208a, -208b, -206, -486 and -499 are specifically expressed in muscle; hence, they have been designated as myomiRs. The analysis of the expression levels of circulating myomiRs represents a powerful tool to investigate miRNA secretion from muscle. Indeed, after the pioneering study by Baggish *et al*. [7], using other exercise protocols, researchers have shown the modulation of specific myomiRs in blood. For example, it has been demonstrated that serum levels of the muscle-enriched miR-486 decrease after both acute and chronic aerobic exercise [9], while a resistance exercise bout of five sets of 10 repetitions at 70% of maximum strength, with 1 min rest between sets, resulted in an increase in miR-149-3p and a decrease in miR-146a and miR-221 three days after the resistance exercise [10]. Nevertheless, Uhlemann *et al*. [11] reported that after a maximal symptom-limited exercise test and four hours of cycling, the concentration of plasma miR-133 remained unchanged, whereas both a marathon race and eccentric resistance training led to an increase in the plasma levels of miR-133. These increases in miR-133 levels were associated with a significant rise in creatine phosphokinase activity in the plasma after the exercise sessions. Very recently, Banzet *et al*. [12] investigated miRNAs in the plasma of exercising humans in order to determine whether they were affected by eccentric and/or concentric exercise. Plasma levels of circulating miRNAs assessed before and after recovery showed that miR-1, -133a, -133b and -208b were not affected by concentric exercise, whereas they increased after eccentric exercise; conversely, miR-181b and -214 significantly increased after concentric exercise. Taken together, these findings suggest that circulating miRNA levels are modulated in response to exercise, and miRNAs may play important roles in exercise-induced adaptations.

The discovery of circulating miRNAs raises questions regarding their function. Recent studies have suggested that circulating miRNAs could mediate gene expression in target cells such as hormones and cytokines [13–15].

Numerous studies indicate that circulating miRNAs may be protected from degradation by several complementary mechanisms, including their incorporation into secreted membrane vesicles such as microvesicles, exosomes or apoptotic bodies [16–18], or *via* the formation of RNA-binding protein complexes [19].

Vesicles in the human circulatory system were first described over 40 years ago. Extracellular vesicles (EVs) are spherical structures bound by a lipid bilayer, which is similar in its composition to the cell membrane from which the vesicle is derived. These vesicles carry signals either in their limiting membrane or in their interior lumen and can be secreted from various cell types to the extracellular environment both constitutively and in a regulated manner [20,21]. It has been widely demonstrated that EVs can be released by many different types of cells and operate as safe containers mediating inter-cellular communication [22]. Their contents include a variety of cytoplasmic elements, which are also a reflection of their cell of origin. Biochemical and biological techniques have been used to identify the protein, mRNA, miRNA, DNA and lipid contents of EVs [23–25].

Although an association between circulating miRNA and EVs has been demonstrated [26], the mechanisms through which muscle releases miRNAs in plasma have yet to be determined.

In this study, we performed EV purification from the blood of sedentary and physically fit young men to investigate whether muscle can release EVs into the bloodstream and whether these vesicles contain muscle specific miRNAs and/or proteins possibly involved in exercise adaptations. Our results suggest a potential role for circulating EVs as biomarkers of exercise physiology.

## Materials and Methods

### Ethical approval

The experimental protocol was performed according to the Helsinki Declaration of 1975 as revised in 2008 regarding ethical principles for medical research involving human subjects. The project was approved by the scientific committee of the University of Urbino *Carlo Bo* (Approval Number 28507). Written informed consent was obtained from all participants.

### Subjects

Twenty-two male students from the University of Urbino *Carlo Bo* participated in the study. They were chosen on the basis of their answers to questions asked in a simple face-to-face interview, which aimed to determine their fitness level. The authors attempted to choose a range of subjects that could represent the whole fitness level continuum (from poor to superior categories, see [27]). The interview evaluated the subjects’ weekly training frequency, intensity and duration, and allowed us to make an initial screening based on the subjects’ presumed fitness level, which was later confirmed through direct fitness testing (see below). Exclusion criteria were: participation in any regular resistance training program for at least 1 month before the experiment; chronic or recent (≤ 2 weeks) treatment with drugs acting on skeletal muscle; recent (≤ 3 month) history of traumatic muscle injury; history of cardiovascular disease. Eighteen subjects completed the study (see Table 1 for participants' characteristics) and 7 of them were assigned to a subgroup that underwent both the fitness test and an acute exercise bout (see below).

**Table 1:** Characteristics of the 18 male participants.

		age	height	body mass	BMI	FM	HR_max_	VO_2max_	reference
		(years)	(m)	(kg)	(kg/m^2^)	(%)	(b min^-1^)	(ml*kg^-1^ min^-1^)	centile*
mean		26	1.76	70.1	22.7	13.9	187.4	58.1	n.a.
*s*(±)		4.8	0.06	9.0	2.9	5.6	2.4	13.1	n.a.
range	min	20	1.65	57	18.2	4.9	183	37.7	<25
	max	36	1.88	89	28.4	24.3	190	75.1	>99

*Note*: BMI, body mass index; FM, fat mass; HR_max_, maximum heart rate; VO_2max_, maximal oxygen consumption; n.a., not applicable; *s*, standard deviation. HR_max_ and VO_2max_ resulted from the fitness test (see text).

*age-related centile reference values from the American College of Sports Medicine [27].

### Measures

#### Anthropometrics

Weight (barefoot, to nearest 0.5 kg) and height (barefoot and head in the Frankfurt plane, to nearest 0.01 m) were measured and BMI (kg/m^2^) was calculated. Skinfold thickness (right subscapular, biceps, triceps, and supra-iliac sites, to the nearest 0.001 m) were measured in triplicate using the Harpenden skinfold caliper (Baty international) according to the recommendations of Lohman *et al*. [28]. Measurements were averaged and fat mass calculated according to Davidson *et al*. [29].

#### Fitness test

Fitness testing was performed on a treadmill (Technogym) and consisted in a graded running test followed by an incremental verification-stage to exhaustion [30]. After a short warm-up, the test was administered in two parts. In the first part, treadmill belt speed was increased by 1 km h^-1^ at the end of each 3-min stage while treadmill grade was maintained constant at 1%. Running speed in the first stage ranged from 8 km h^-1^ to 13 km h^-1^ according to the participant’s fitness level. Subjects completed a minimum of 5 and a maximum of 9 stages. The treadmill was stopped when the subject would only have been able to complete one more stage if he had been required to continue running. The in-between resting period consisted in a 5-min cool-down followed by 10-min rest. In the second part of the protocol, running speed was kept constant and calculated by subtracting 2 km h^-1^ from the speed reached in the last stage of the first part of the test. The treadmill grade was increased by 1% after each 1-min stage until the subject reached volitional exhaustion. During this phase strong verbal encouragement was provided. Throughout the whole test, heart rate (HR) was monitored continuously with a HR rate monitor (Polar Electro) and pulmonary gas exchange was sampled breath-by-breath with a metabolic cart (Vmax, Carefusion). Before starting each test, the gas analyzers of the metabolic cart were calibrated using known ambient-air and sample gas reference (16% O_2_, 5% CO_2_), while the turbine flow meter was calibrated with a syringe of known volume (3 liters). In accordance with Howley *et al*. [31], subjects were considered to have reached their maximal oxygen consumption (VO_2max_) when one or more of the following criteria were met: i) leveling off of oxygen consumption with increasing workload; ii) respiratory exchange ratio values >1.1; and iii) an HR of at least 90% of the age-predicted maximum. VO_2max_ was defined as the highest 20s average during the final minute of the verification-stage.

### Acute exercise protocol

A subgroup of the enrolled subjects, characterized by superior VO_2max_, engaged in an acute aerobic exercise session. The warm-up consisted in 15 minutes of running on a treadmill (constant 1% grade) with progressive increases in speed (until 2 km h^-1^ less than the hypothesized exercise speed) and was followed by a 5-min resting period. The 40-minute, vigorous-intensity, aerobic exercise bout started in the early morning (9:20 am) with the treadmill set at a 1% grade. Exercise intensity was set at approximately 80% of the VO_2max_ and was monitored using HR. The HR target was derived from data sampled in the first phase of the fitness test, as the average HR recorded in the last 30s of the 3^rd^ minute of the stage that elicited the steady-state oxygen consumption closest to 80% of the VO_2max_. The treadmill speed was adjusted accordingly whenever the difference between exercising HR and the HR target was greater than ±5 beats min^-1^.

### Blood sampling

Participants’ blood was sampled at rest 1 hour before the fitness test with the exception of the subject assigned to the acute exercise subgroup, whose blood was sampled both at rest 1 hour before (baseline) and 1 hour after (post-exercise) the end of the aerobic exercise bout.

Blood was drawn in fasting condition from the antecubital vein and it was collected into tubes containing EDTA for plasma samples (12 ml) (Becton Dickinson). Plasma was separated immediately by centrifugation (1000 g, 4°C, 10 min). Subsequently, 350 μl aliquots were frozen at -80°C while 5 ml of plasma were freshly used for extracellular vesicle purification.

### Extracellular vesicle isolation

EV isolation was carried out following the guidelines reported in Turchinovich *et al*. [14]. In brief, plasma was first cleared by centrifugation for 15 min at 1000*g* to eliminate cell contamination. Supernatants were further centrifuged for 20 min at 12,000*g* and subsequently for 20 min at 18,000–20,000*g*. The resulting supernatants were filtered through a 0.22 μm filter and then EVs were pelleted by ultracentrifugation at 110,000*g* for 70 min. The EV pellets were washed in 13 ml PBS, pelleted again and resuspended in PBS.

### Optiprep density gradient separation

A discontinuous iodixanol gradient was used to float the EVs purified from plasma. Iodixanol 40% (w/v), 20% (w/v), 10% (w/v) and 5% (w/v) solutions were prepared diluting OptiPrep (60% (w/v) aqueous iodixanol (Axis-Shield) with 0.25 M sucrose/10 mM Tris, pH 7.5 and gradient was performed as reported in Tauro *et al*. [32]. EV pellet purified from plasma was resuspended in 500 μl of 0.25 M sucrose/10 mM Tris, pH 7.5 and overlaid onto the top of the gradient. Centrifugation was performed at 110,000g overnight at 4°C. Twelve individual 1 mL gradient fractions were collected, diluted with 13 ml of PBS and then centrifuged at 110,000g for 1 h at 4°C and resuspended in PBS. Fractions were monitored for the expression of exosomal marker TSG101 and the muscular marker α-Sarcoglycan by Western blotting. The density of each fraction was determined by absorbance at 244 nm of 1:10,000 diluted fractions as reported [32].

### Isolation of exosomes using alpha-sarcoglycan immunoaffinity capture beads

Exosome-Dynabeads Streptavidin (Life technologies) in PBS + 0.1% BSA were mixed with biotinylated anti-alpha-sarcoglycan (clone AD1/20A6 Monosan) according to the manufacturer’s instructions. EVs were purified from 10 ml human plasma following the serial ultracentrifugation protocol. The obtained pellet was resuspended in PBS + 0.1% BSA and was pre-incubated with Exosome-Dynabeads Streptavidin for 2 h at 4°C with gentle rotation to reduce non-specific binding. The beads were harvested using a magnet and the supernatant was retained and incubated with the prepared anti-alpha-sarcoglycan immunoaffinity capture Dynabeads overnight at 4°C with a gentle rotation. Bound EVs were directly lysed with Qiazol (Qiagen) for miRNA and protein purification.

### miRNA quantification

Total RNA was extracted from the EV pellets, purified using the miRNeasy Mini kit (Qiagen) according to the manufacturer’s instructions, and finally contaminant DNA was digested with DNase I enzyme (Ambion). For the identification of mir-206 in EV pellets from Density Gradient separation and Immuno-capturing, cDNA was synthetized using the TaqMan MicroRNA Reverse Transcription Kit (Life Technologies). An Applied Biosystems StepOne Plus Real-Time PCR device was used to amplify cDNA using fluorescently labeled Taqman probe and primer sets. Taqman primer/probes for quantification were used as follows (Life Technologies): hsa-miR-16 [miRNA ID: 000391], hsa-miR-206 [miRNA ID: 000510], ce-miR-39 [miRNA ID: 000200].

The miScript Reverse transcription kit (Qiagen) was used for the quantification of miRNAs contained in EVs in response to exercise. The quantitative PCRs were performed with two microliters of cDNA in a StepOne Plus Real-Time PCR (Applied Biosystems) using the miScript SYBR Green PCR Kit (Qiagen) and miScript Primer Assays (Qiagen) specific for human miR-1, miR-133a, miR-133b, miR-206, miR-181a-5p, mir-499 and miR-146a. Furthermore, we performed the expression analysis of the previously described stable plasma miRNAs, miR-16, miR-191 and miR-24 [26]. The real-time PCR conditions were: 95°C for 10 min followed by 45 cycles of three-steps at 95°C for 15 sec, 55°C for 15 sec, and 70°C for 30 sec for miScript Primer Assays (Qiagen) and 95°C for 10 min followed by 45 cycles of two-steps at 95°C for 10 sec, 60°C for 1 min for Taqman primer/probes (Applied Biosystems). Fractional quantification cycle (Cq) values were determined by the *Cy0* method [33]. Quantification was performed according to the ΔCq method [34], miR-16 or the exogenously added spike-in cel-miR-39 were used as a reference.

### Western blotting analysis

Proteins were extracted from the organic phase after phenol separation of RNAs containing aqueous phase following Qiagen User Protocol RY16 May-04. The obtained protein pellet was resuspended in ISOT buffer (8 M urea, 4% CHAPS, 65 mM DTE, 40 mM Tris base) and sonicated for 5 s on ice. After centrifugation at 18,000 *g*, protein concentration was determined by the Bradford assay [35]. For electrophoresis, samples were mixed with the Laemmli sample buffer 4X (1:4 ratio) and loaded onto 12% SDS-PAGE gels. The proteins were then blotted to a PVDF membrane (GE Healthcare). Primary antibodies were used against Tsg101 (1:2,000 dilution, clone 4A10 Abcam) and alpha-sarcoglycan (1:300 dilution, clone H-82 Santa Cruz). Primary antibodies were incubated overnight at 4°C followed by washing and the application of secondary HRP conjugated antibody (Pierce). Immune complexes were visualized using the Supersignal Dura reagent (Pierce), and the obtained auto-radiographic films were quantified using ImageJ software.

### Flow Cytometric characterization of Extracellular Vesicles

EVs were purified from human plasma by differential centrifugation as reported above. The EVs were resuspended in PBS + 0.1% BSA and then stained with an anti-CD81 PE (clone BD Pharmingen, clone JS-81) or anti-alpha-sarcoglycan (clone AD1/20A6 Monosan) and labelled by goat anti-mouse (GaM) FITC. The cytometric analyses were performed by gating events smaller than 1 mm. Size beads (Ø 1–2 mm Polysciences Invitrogen, and Ø 5.2 mm DakoCytoCount beads) were used to establish the proper gate for events smaller than 1 mm, which include EVs, and to obtain single platform absolute counts [36].

### Statistics

**Fig 1:**
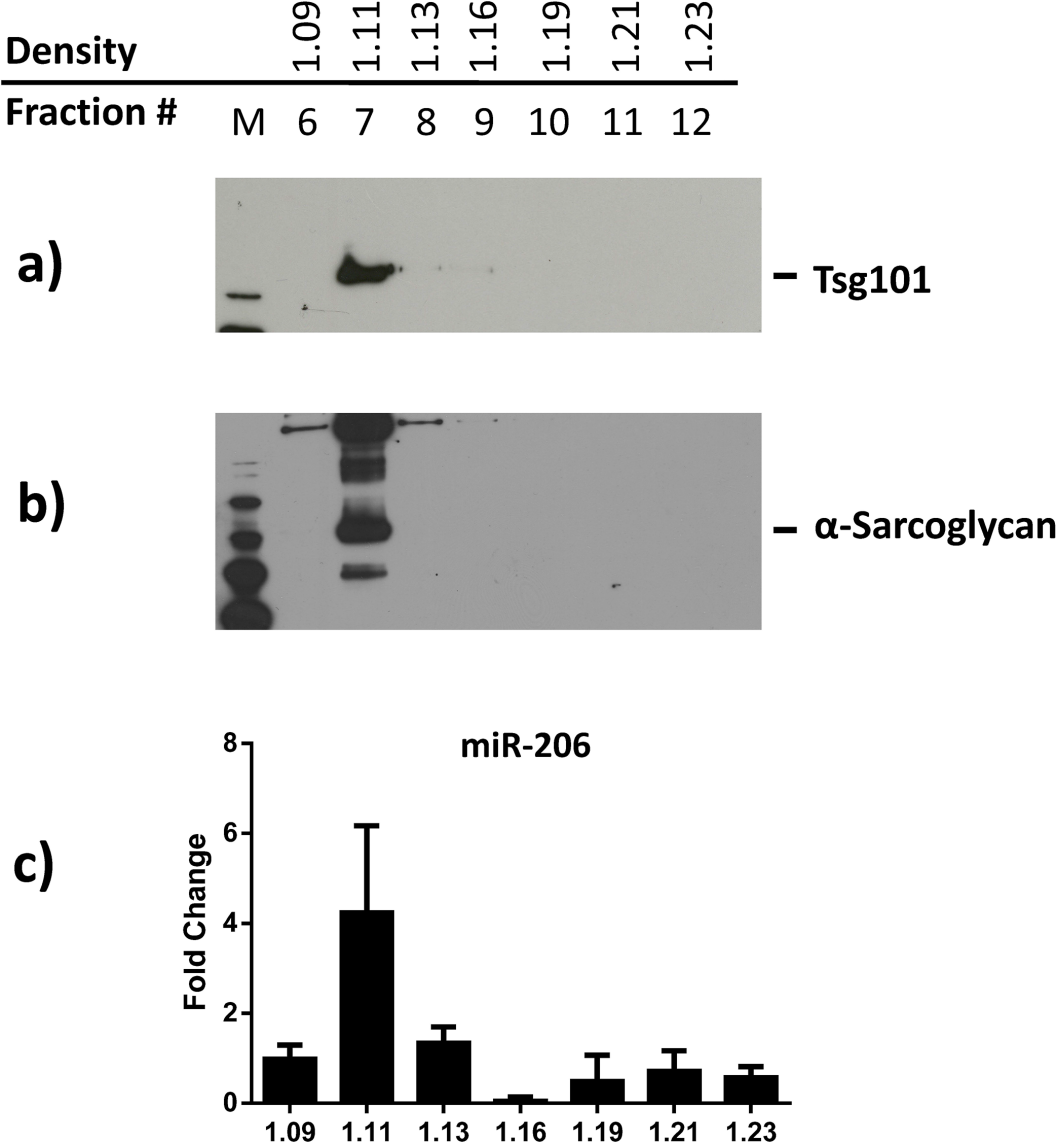
Density gradient separation of plasma EVs. Plasma EVs were purified using the serial ultracentrifugation protocol. The obtained pellet was then further separated using the Optiprep iodixanol density gradient. Fractions containing total EVs were identified by Western blot analysis with antibodies against Tsg101 (a) while EVs originating from muscle tissue were identified by the anti-SGCA (b). MiR-206 expression levels were quantified from each fraction using a specific TaqMan MicroRNA probe, expression levels were normalized versus the spike-in cel-miR-39 exogenously added and reported as fold change compared to the fraction 6 (c).

**Fig 2:**
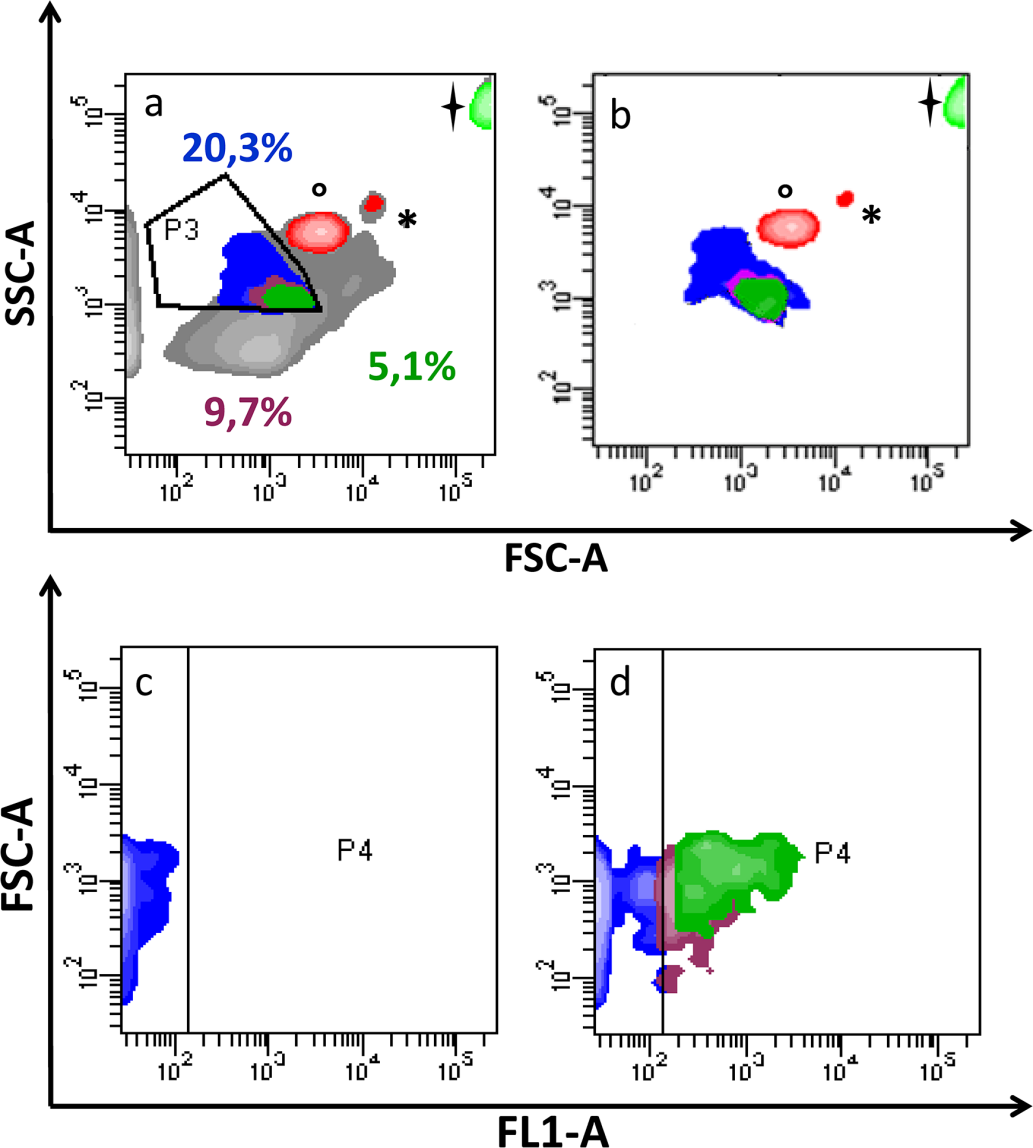
Contour plot of plasma EVs. EVs were evaluated (P3) using size beads, identified by °(1 μm), *(2 μm) and (5.2 μm) (a). This gate strategy was performed to define the proper gate for events smaller than 1μm (P3), including MVs. Selected events are shown in contour plots (c) and (d), from samples labelled by goat anti-mouse (GaM) FITC (FL1-A) and anti-SGCA+ GaM FITC. P4 represents the area of FL1 positivity, drawn taking into account cluster distribution into the “negative” sample (MVs labelled with GaM only). Percentages are related to the following subpopulations: Purple: SGCA+ events; Blue: events less than 1 μm; Green: CD81^+^/SGCA^+^ events, shown in Fig 2A and 2B.

Subject characteristics and VO_2max_ data are reported as means and standard deviation (*s*). Data were tested for normality using the Kolmogorov-Smirnov test. MiRNA data are presented as means ± standard error of the mean (Figs 1 and 2). Correlation analyses were performed using Pearson’s method. Quantitative measures of EV-miRNA levels during exercise are presented as bar and whisker plots where horizontal lines denote median, boxes denote 25% and 75% percentile confidence intervals, and error bars reflect maximum and minimum values (GraphPad Prism 6.0).Paired Student t-tests were used to test for differences between Rest and Post-Exercise groups; statistical significance was established at *P<*0.05 (Statistica 5.0, Statsoft).

## Results

### Characterization of muscle EVs from plasma

EVs were isolated from the plasma of healthy donors using serial ultracentrifugations followed by the separation of the obtained vesicles by density gradient to avoid the co-precipitation of soluble proteins. Fig 1 shows the western blot analysis of 1 mL fractions obtained using a discontinuous 5–40% OptiPrep density gradient separation. As shown in Fig 1A, plasma-derived EVs, identified through the quantification of the exosomal marker TSG101, were enriched at a buoyant density of 1.11 g/mL, as previously described [37]. The 1.11 g/mL density fraction also resulted positive to SGCA immuno-staining (Fig 1B). The positivity to anti-SGCA suggests the presence in the plasma of EVs that originated from muscle tissues. The OptiPrep density fractions were further analyzed for the presence of miR-206, a miRNA specifically expressed in muscle tissues [38]. Fig 1C clearly shows that, in addition to TSG101 and SGCA, miR-206 was enriched in the 1.11 g/mL density fraction. These data suggest that miR-206 could be secreted within a sub-population of the isolated EVs and also provide further evidence that these EVs originate from muscle tissues.

### Cytofluorimetric analysis of plasma EVs

**Fig 3:**
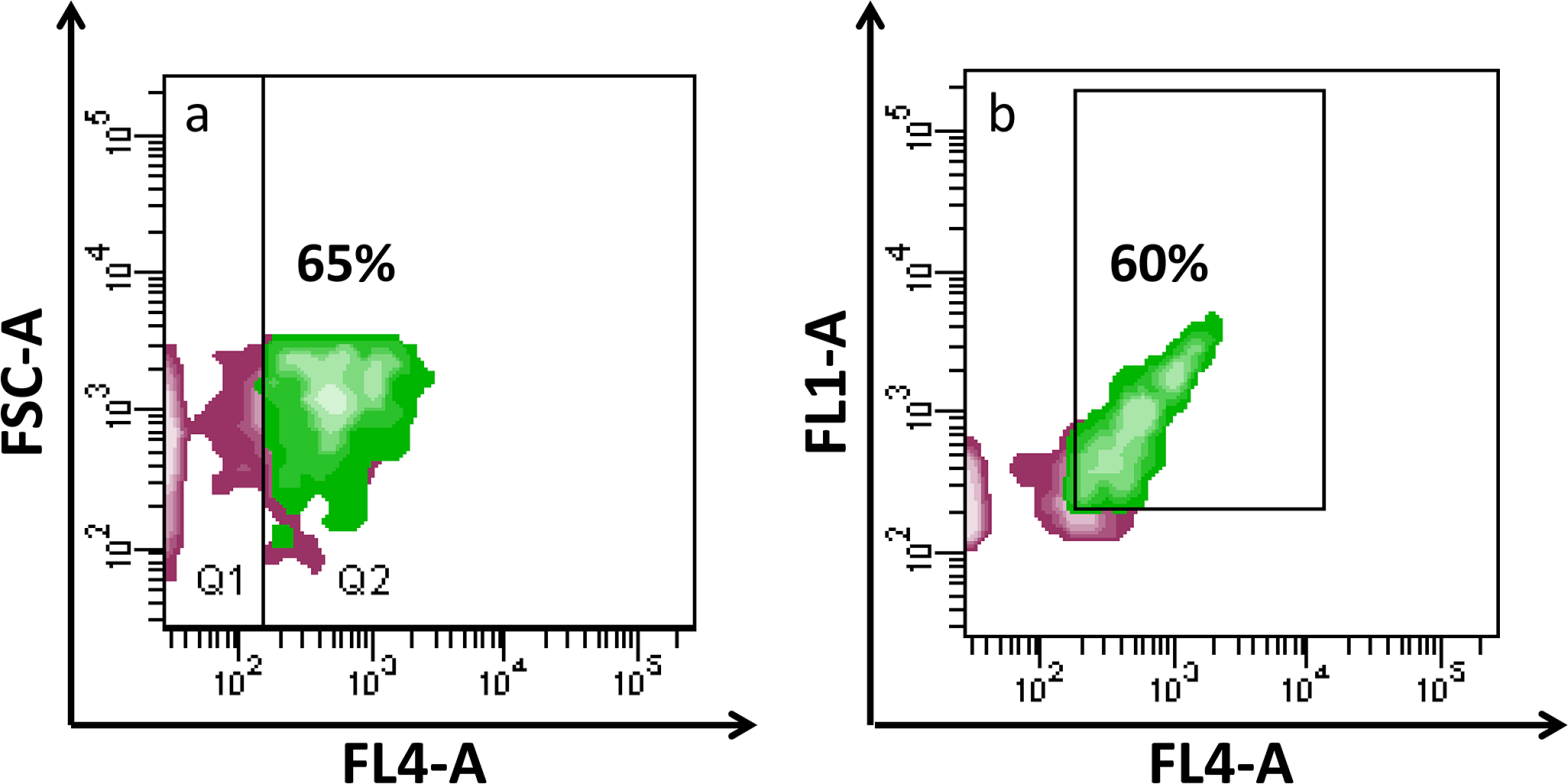
Contour plots of size-selected EVs. Briefly, green clusters are those gated on the basis of their smaller size and SGCA positivity (previous contour plots: green cluster) and visualized for: a) for CD81 (FL4-A) and FSC localization; b) CD81 (FL4-A) and SGCA positivity (FL1-A), contour plot emphasizing co-expression of both markers.

The presence of muscle-derived EVs in circulation was further investigated using flow cytometry. EVs separated by ultracentrifugation were investigated for their expression of CD81, a well-established exosomal surface marker [36], and for the muscle marker SGCA. The cytometric data reported in Fig 2A show the presence of EVs <1μm in diameter positive for anti-SGCA (green area), these SGCA^+^ EVs are highlighted in Fig 2D (FL1 = SGCA-FITC). The P3 gate setting allowed us to estimate that muscular EVs isolated from plasma represented about 5% of the total (Fig 2A). In the contour plot in Fig 2B, only P3 (best characterized EV sub-populations) and bead events are shown, after using a gating process able to purge aggregates and putative debris. The contour plot in Fig 2C corresponds to the negative control (EVs labelled with goat anti-mouse FITC only), whereas the plot in Fig 2D shows a significant shift of the fluorescence signal after the addition of the anti-SGCA antibody, highlighting the anti-SGCA^+^ sub-population in circulating EVs. To further characterize the anti-SGCA^+^ sub-population the presence of CD81 was investigated. Fig 3 and 3B show that 60–65% of the muscular EVs were also positive for anti-CD81, appearing as purple events in all the contour plots. Specifically, Fig 3A shows CD81 positivity coupled with particle size, whereas Fig 3B highlights the co-expression of CD81 and SGCA.

### Immuno-capturing of muscle-derived EVs from plasma

**Fig 4:**
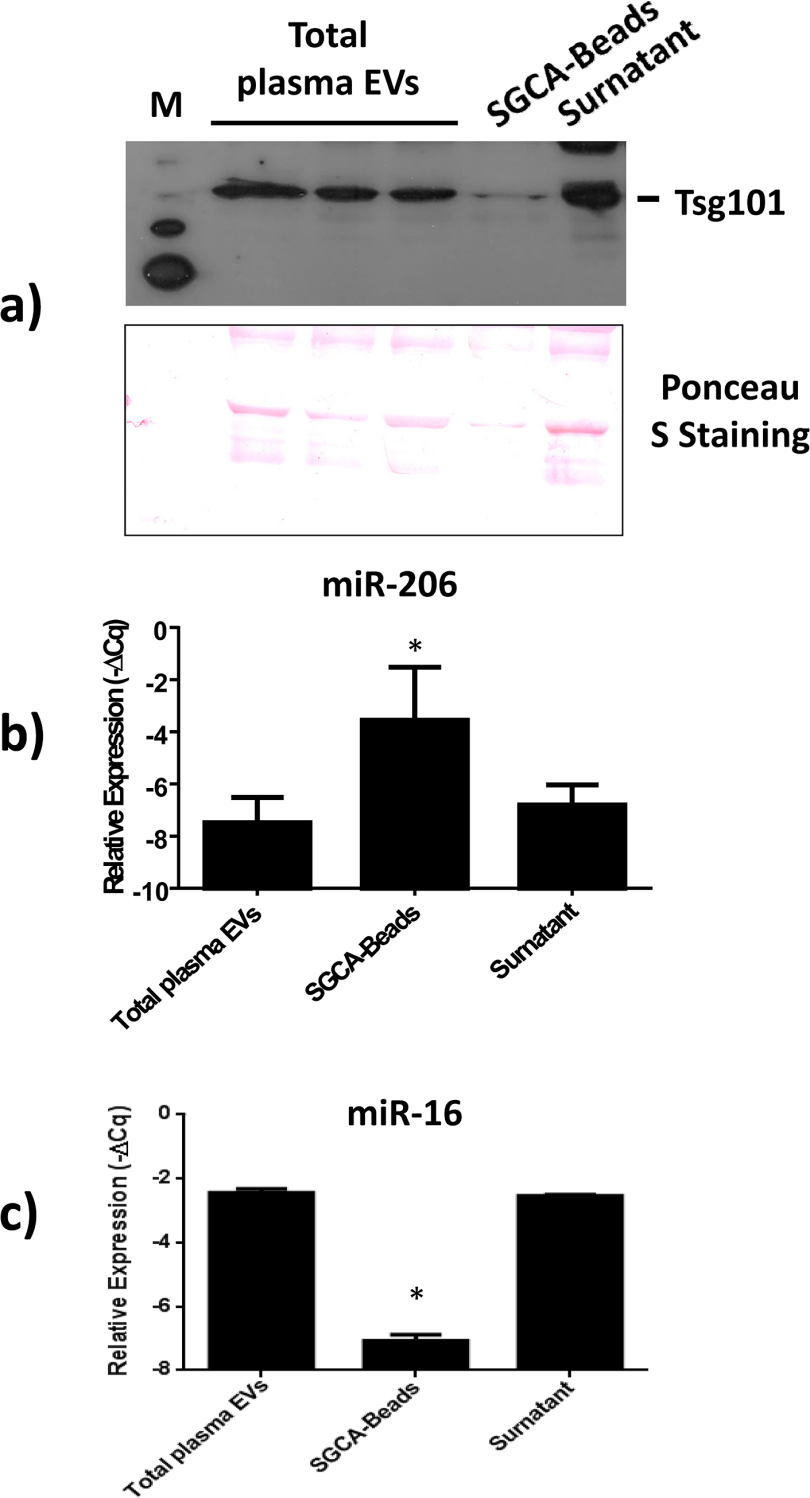
Isolation of alpha-sarcoglycan^+^ EVs from plasma using immunoaffinity capturing. Anti-alpha-sarcoglycan antibodies were conjugated to magnetic beads to isolate muscle EVs from plasma. Western blot analysis confirmed the presence of the exosomal marker Tsg101 in isolated EVs. Ponceau S Staining has been used as loading control (a). MicroRNA quantifications showed an increase in the miR-206/miR-16 ratio in the SGCA^+^ sub-population of EVs (SGCA-Beads) compared to total (total plasma EVs) or uncaptured EVs (Supernatant). MiR-206 expression levels were normalized versus the endogenous reference miR-16 and expressed as -ΔCq (where ΔCq = Cq_miR-206_-Cq_miR-16_) (b). Moreover, the quantification of miR-16 ratio in the SGCA^+^ sub-population of EVs compared to total or uncaptured EVs shows that SGCA-conjugated beads retained about 2–5% of the total amount of EVs, miR-16 expression levels were normalized versus the spike-in reference cel-miR-39 and expressed as -ΔCq (where ΔCq = Cq_miR-16_-Cq_cel-miR-39_) (c). Asterisks denote significant changes (*p*<0.05).

Finally, anti-SGCA antibodies were conjugated to magnetic beads in order to isolate muscle EVs from plasma to directly investigate whether the SGCA^+^ sub-population of circulating EVs is enriched in muscular miR-206. Circulating EVs were purified from 10 mL of plasma as previously described, and the obtained pellet was resuspended in PBS containing 0.1% BSA and incubated overnight with anti-SGCA conjugated magnetic beads. Western blot analysis confirmed the presence of TSG101^+^ EVs in the controls and in the immuno-captured EVs (Fig 4A). As expected, microRNA quantification revealed an increase in the miR-206/miR16 ratio in the SGCA^+^ sub-population of EVs compared to total or uncaptured EVs (Fig 4B). These data further support the hypothesis that the SGCA^+^ sub-population of EVs could originate from muscle tissue. Moreover, the comparison of the miR-16 levels within the EVs, obtained from magnetic beads, with the miR-16 levels in the corresponding unbound EVs, revealed that the SGCA-conjugated beads retained about 2–5% of the total amount of EVs (Fig 4C), which is consistent with the flow cytometric data.

### Correlation between EV miRNAs and aerobic fitness

**Fig 5:**
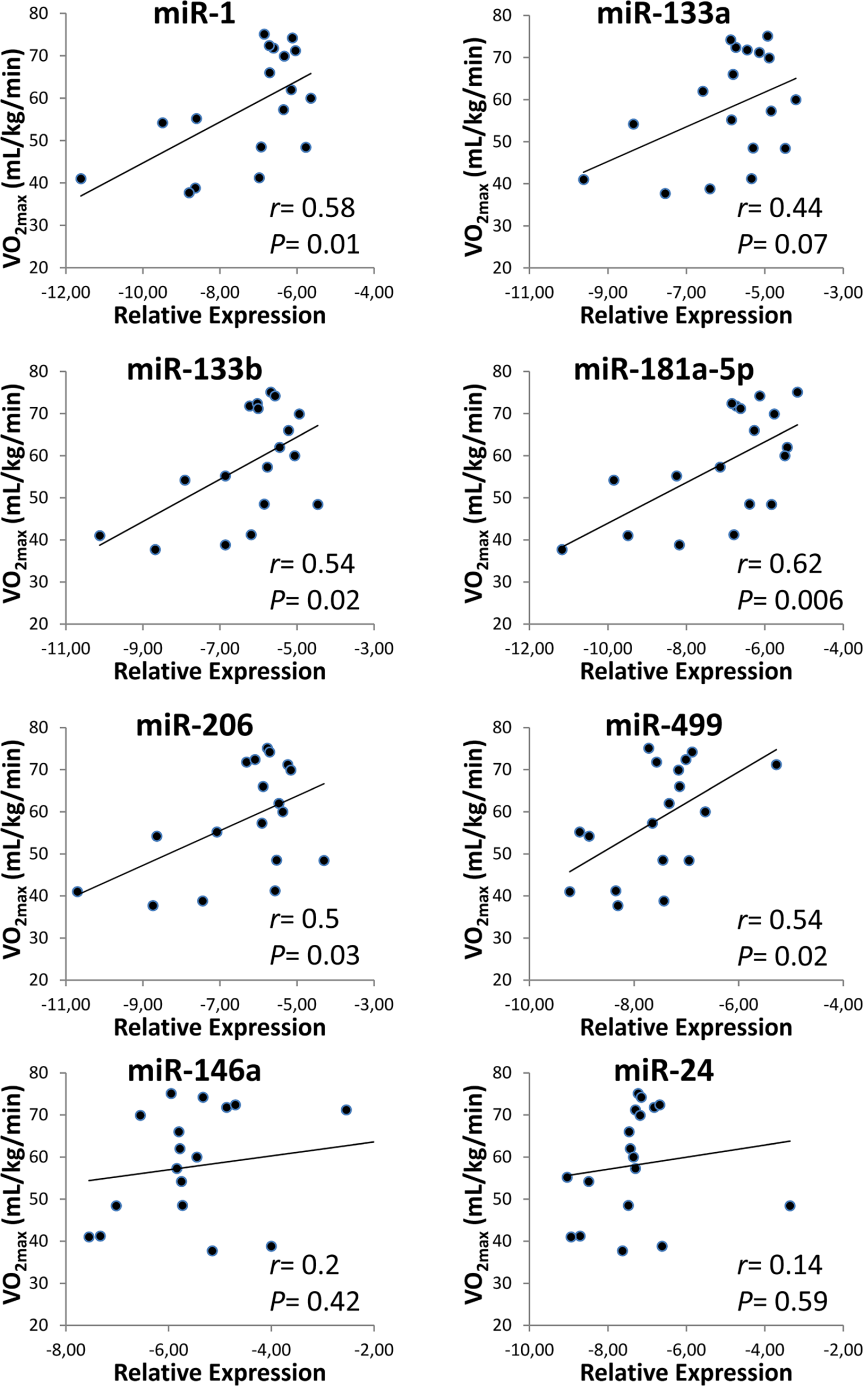
Expression levels in specific EV miRNAs correlate with aerobic fitness. For each volunteer (*n* = 18), baseline miRNA levels from plasma EVs under resting condition were assigned to the corresponding aerobic fitness level estimated as VO_2max_. MiRNA expression levels were reported as -ΔCq (where ΔCq = Cq_miR-206_-Cq_miR-16_). A direct significant correlation (*r* = correlation coefficient) was observed between levels of miR-1, miR-133b, miR-206, miR-499 and miR-181a-5p (baseline) and VO_2max_. MiR-24 expression; which is stably expressed in blood, is the negative control.

To test whether circulating EV-encapsulated miRNAs can be used to gain insights into muscle adaptations we investigated the modulation of the miRNA expression levels contained within blood-derived EVs in relation to aerobic fitness levels. The following miRNAs: miR-1, miR-133a, miR-133b, miR-206 and miR-499, specifically expressed in muscle, and the well characterized miR-146a and miR-181a-5p were analyzed in circulating EVs from 18 healthy subjects representing a wide range of aerobic fitness levels (Table 1). In addition, the miRNAs miR-16 and miR-24, which are stably expressed in blood [26], were used for endogenous normalization and as a negative control, respectively. A significant positive correlation was found between aerobic fitness (measured as VO_2max_) and miR-1, miR-133b, miR-206, miR-499 and miR-181a expression levels from purified EVs (Fig 5). These data suggest that miRNAs within plasma EVs could be used as aerobic fitness biomarkers and could provide useful information on muscle homeostasis.

### Modulation of circulating EV miRNA in response to acute exercise

The presence of miRNA in the bloodstream could be due to active export systems and/or passive leakage through the plasma membrane following cell damage or death. Furthermore, export systems could involve different miRNA carriers such as EVs, HDL [39] and AGO-proteins that ensure protection from extracellular nucleases stabilizing circulating miRNAs.

In response to muscle contraction, both active and passive miRNA release could occur, contributing to the levels of circulating miRNAs; however, several studies have highlighted that circulating muscle-enriched miRNAs mainly increase in response to muscle damage [11,12]. Importantly, circulating free-miRNAs could have different biological roles than miRNAs wrapped in lipid membranes because there is no clear evidence to date of the internalization of extracellular free-miRNAs. On the other hand, there is a great deal of evidence demonstrating the internalization and regulation activity of miRNAs contained in EVs [40,41].

**Fig 6:**
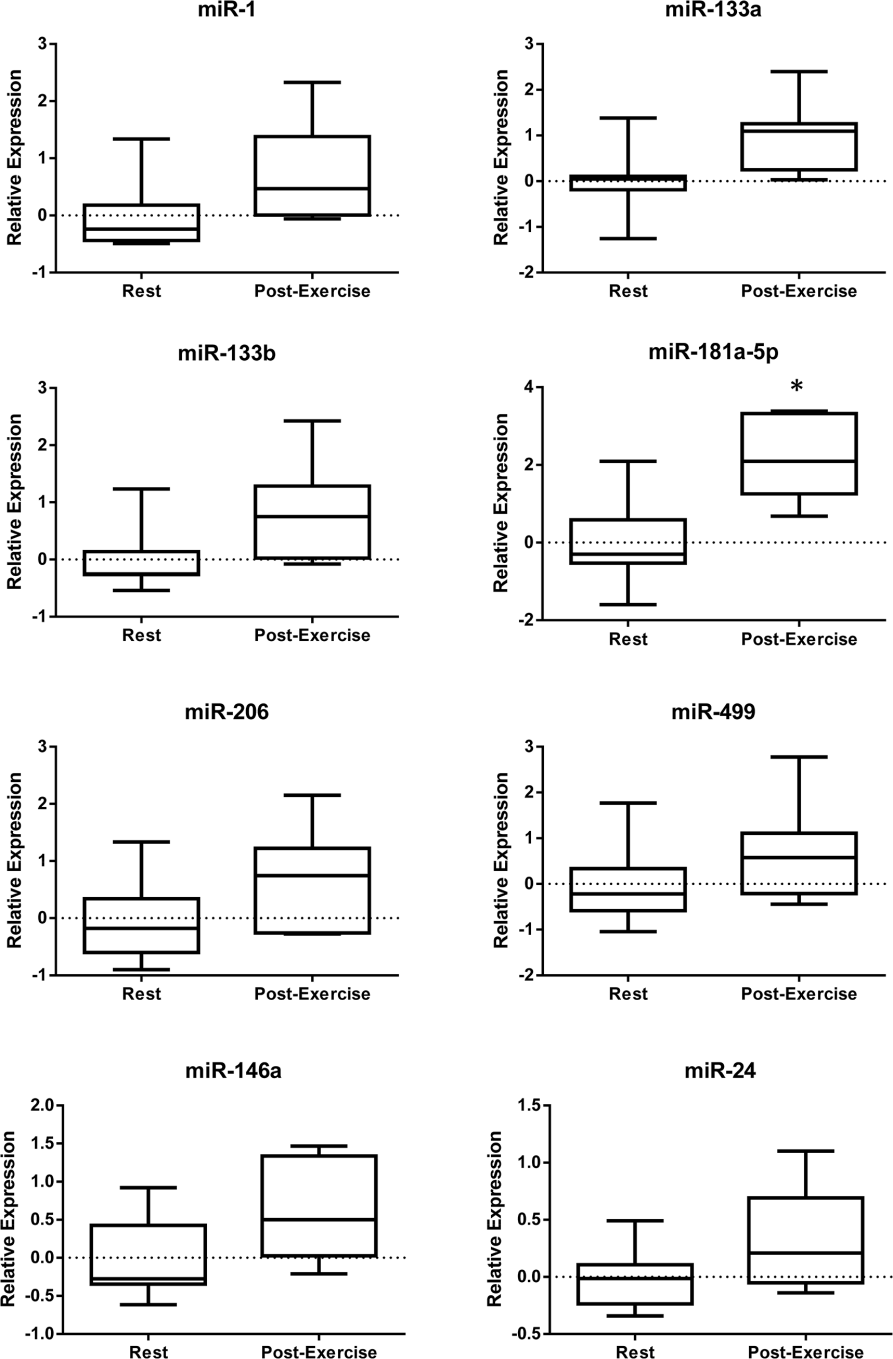
Modulation in circulating EV miRNAs in response to an aerobic exercise bout. The expression levels of miR-1, -133a, -133b, -146a, -181a-5p, -206, and -499 were evaluated in the circulating EVs of healthy volunteers (*n* = 7) at rest (Rest) and 1-hour after a 45-min aerobic exercise bout at 65% VO_2max_ (Post-Exercise). MiRNA expression levels were reported as -ΔΔCt obtained subtracting the ΔCq at rest (where ΔCq = Cq_miR-206_-Cq_miR-16_) from the ΔCq Post-Exercise. MiR-24 is the negative control. Asterisks denote significant changes (*p*<0.05).

The above-mentioned data show that the release of miRNAs into the bloodstream could be carried out, at least in part, by means of EVs; hence, we investigated the release of some key miRNAs contained in circulating EVs after acute aerobic exercise. Blood samples were collected at rest, immediately before exercise and one hour after acute aerobic exercise. Circulating EVs were purified by ultracentrifugation and the miRNA expression levels were quantified. As reported in Fig 6, miR-181a-5p was significantly up-regulated after exercise while miR-133b showed only a borderline increase. These data support the notion that circulating EV miRNAs could provide us with insights into the molecular mechanisms driving adaptation to exercise.

## Discussion

Recent studies have demonstrated that muscle miRNAs (MyomiRs) are present in the serum and plasma in a variety of physiological and pathological conditions [26,42]. Although an association between circulating miRNA and EVs such as microvesicles, exosomes or apoptotic bodies has been demonstrated [26,43,44], the mechanisms through which muscle releases miRNAs in plasma have yet to be determined.

The main aim of this study was to investigate whether muscle tissue releases EVs carrying microRNAs in the bloodstream under physiological conditions. To achieve this goal, we used a combination of three techniques: cytometric analysis, density gradient separation and immuno-capturing. The role of physical activity and acute exercise in the modulation of the miRNAs loaded into EVs was also evaluated.

The obtained results provide us with two important insights. Firstly, muscle tissues release EVs into the bloodstream and secondly, the MyomiRs detected in circulation are at least in part engulfed in muscle-derived EVs. Interestingly, MyomiR levels of the circulating muscle-derived EVs seem to be mainly affected by aerobic capacity, but also by a single bout of aerobic exercise.

Current research on exercise-induced circulating miRNAs does not describe the mechanisms through which miRNAs are secreted. In the extracellular environment, miRNAs could be stabilized by EVs (such as exosomes, microvesicles or apoptotic bodies), HDL/LDLs, or protected by RNA-binding proteins (reviewed in [14,45]).

Our team has already demonstrated that the muscle cell line C2C12 releases exosome-like vesicles in conditioned medium [46]. Furthermore, two key studies from separate groups have recently shown that muscle cells release exosomes carrying bio-active miRNAs [47,48]. The data reported herein confirm these results and provide further insight showing the presence of EVs released by muscle tissue in plasma. Specifically, our results show that circulating EVs present some characteristics typical of exosomes (buoyant density constant and positivity for CD81 and TSG101) and more importantly, a small fraction of these EVs are SGCA-positive and enriched in miR-206 suggesting their muscle cell origin. To the best of our knowledge, these data represent the first evidence that muscle releases EVs into the bloodstream. Moreover, cytometric and immuno-capturing data suggest that muscle-derived EVs in circulation account for 1–5% of the total circulating EVs, which is in agreement with findings suggesting that the majority of blood EVs derive from immune system cells, the endothelium and platelets. Although our results support the presence of muscle-derived EVs in the bloodstream, their absolute amount should be carefully considered. In fact, after being released, SGCA-positive EVs could share their content, making quantification difficult.

Recent studies have reported an association between physical exercise and the modulation of circulating miRNAs. For example, a pioneering study by Baggish *et al*. [7] reported a positive correlation between miR-146a and VO_2max_ levels and more recently Bye *et al*. [49] found that miR-21, miR-210, and miR-222 were higher in the low VO_2max_-group than in the control group.

Unlike previous studies, our study focused on miRNAs packaged in EVs and showed a positive correlation between aerobic fitness and MyomiRs. Although the investigation of circulating miRNA is a powerful tool to identify new biomarkers, it is difficult to associate a free-miRNA with its tissue of origin. To determine whether muscle tissue could be one of the main sources of exercise-induced circulating miRNAs, several investigations have focused on the quantification of circulating miRNAs whose expression is enriched in muscle. However, these studies have yielded conflicting results. For example, following a single bout of cycling miR-1, miR-133a, miR-133b, and miR-181a levels were found to be increased in the skeletal muscle tissue of untrained subjects [50]; however, evidence based on plasma miRNAs showed that all muscle-enriched miRNAs (miR-1, miR-133a, miR-133b, miR-206, miR-208b, and miR-499) remained unchanged after exercise [7,9].

On the other hand, very recently Nielsen *et al*. [51] reported that after acute aerobic exercise, circulating levels of miR-1, miR-133a and miR-133b were increased.

These inconsistencies may be due to differences in the exercise protocols such as the duration of the exercise. Indeed after a marathon, specific miRNAs originating from skeletal muscle (miR-1, miR-133a), cardiac muscle (miR-208a), and inflammatory processes (miR-146a) were robustly up-regulated [52]. Moreover, different mechanisms of miRNA secretion occur in response to different types of exercise. The results reported herein show a significant increase in miR-181a-5p and miR-133b and only a tendency to increase for the other MyomiRs in circulating EVs after acute aerobic exercise. These data are in line with recent studies which have shown an increase in circulating MyomiRs in response to acute aerobic exercise [51,52]; however, these studies did not address the question of which type of carrier transports miRNAs. This is a very important issue because miRNAs engulfed in lipid carriers can be internalized within the target cell and regulate gene expression [53], whereas it is still unclear if this is also true for free-miRNAs.

Interestingly, our data show that the increase in miR-133b (muscle-specific) and miR-181a in circulation following acute exercise stems, at least in part, from active secretion mechanisms, and is not only linked to non-specific leakage due to tissue damage as previously suggested [11,12].

Moreover, studies performed on skeletal muscle have shown increased miR-133b expression levels after an acute bout of endurance exercise, and this up-regulation has been linked to skeletal muscle remodeling and maintenance [50]. Taken together, this evidence suggests that an active muscle can release into the extracellular environment EVs containing miRNAs mediating cell-to-cell communication potentially involved in muscle repair, regeneration and remodeling.

The present finding, that muscle releases EVs into the bloodstream, opens up a new field of research on the secretory activity of this organ. Indeed, a growing body of evidence demonstrates that EVs can act as intercellular communication devices acting as safe vesicular carriers of molecules such as receptors, transmembrane proteins, kinases, mRNA, miRNA, long non-coding RNA, DNA and lipids [54–56]. In light of this evidence, we should expand the current concept of muscle factors beyond soluble factors such as myokines, and also consider EVs. Finally, in addition to their role in cell–cell communication, recent studies have shown that EVs might be good biomarker candidates. Indeed, a novel concept for biomarkers, called “liquid biopsy,” has been proposed [57,58]. Liquid biopsy would be useful for numerous diagnostic applications and eliminate the need for tissue biopsies.

Investigating the messages contained in EVs sent by muscle tissue to other organs might provide us with information regarding the tissue of origin and this in turn may provide us with useful new biomarkers. Future studies could be designed to thoroughly explore the content of muscle EVs in physiological (i.e. exercise) or pathological (i.e. inactivity, atrophy, diabetes, etc.) conditions. Such investigations might provide insight into the role of EVs, not only as useful biomarkers, but also as regulators of the body’s homeostasis.

## Supporting Information

S1 Fig Original uncropped and unadjusted blot corresponding to Fig 1A. (TIF)

S2 Fig Original uncropped and unadjusted blot corresponding to Fig 1B. (TIF)

S3 Fig Original uncropped and unadjusted blot corresponding to Fig 4A. (TIF)
