# Supplementary figures and images for: Muscle Releases Alpha-Sarcoglycan Positive Extracellular Vesicles Carrying miRNAs in the Bloodstream
Source: PLoS One. 2015 May 8;10(5):e0125094. doi: 10.1371/journal.pone.0125094 (PMC4425492; doi:10.1371/journal.pone.0125094)

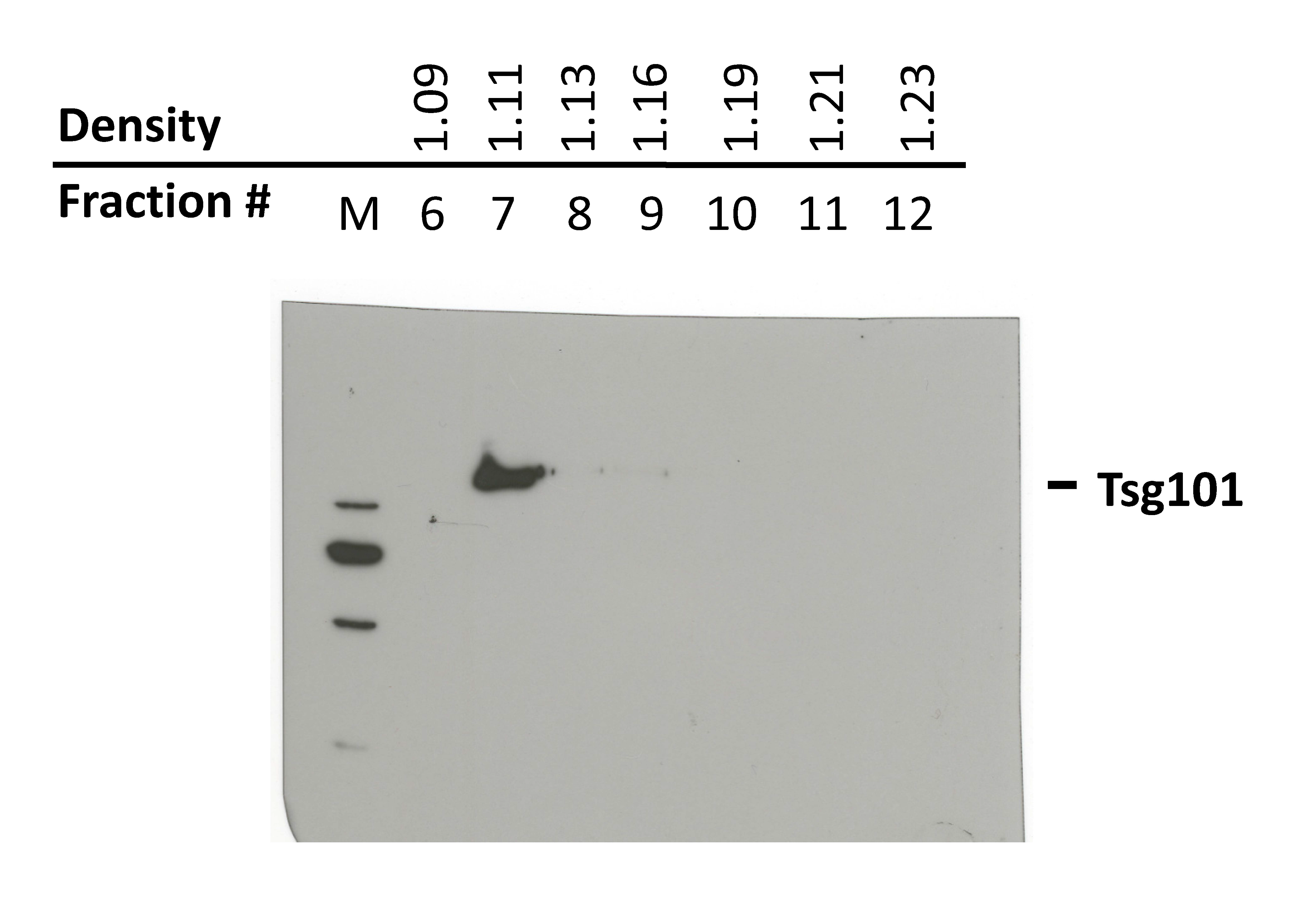

Supplement: S1 Fig — (TIF) [file pone.0125094.s001.tif]

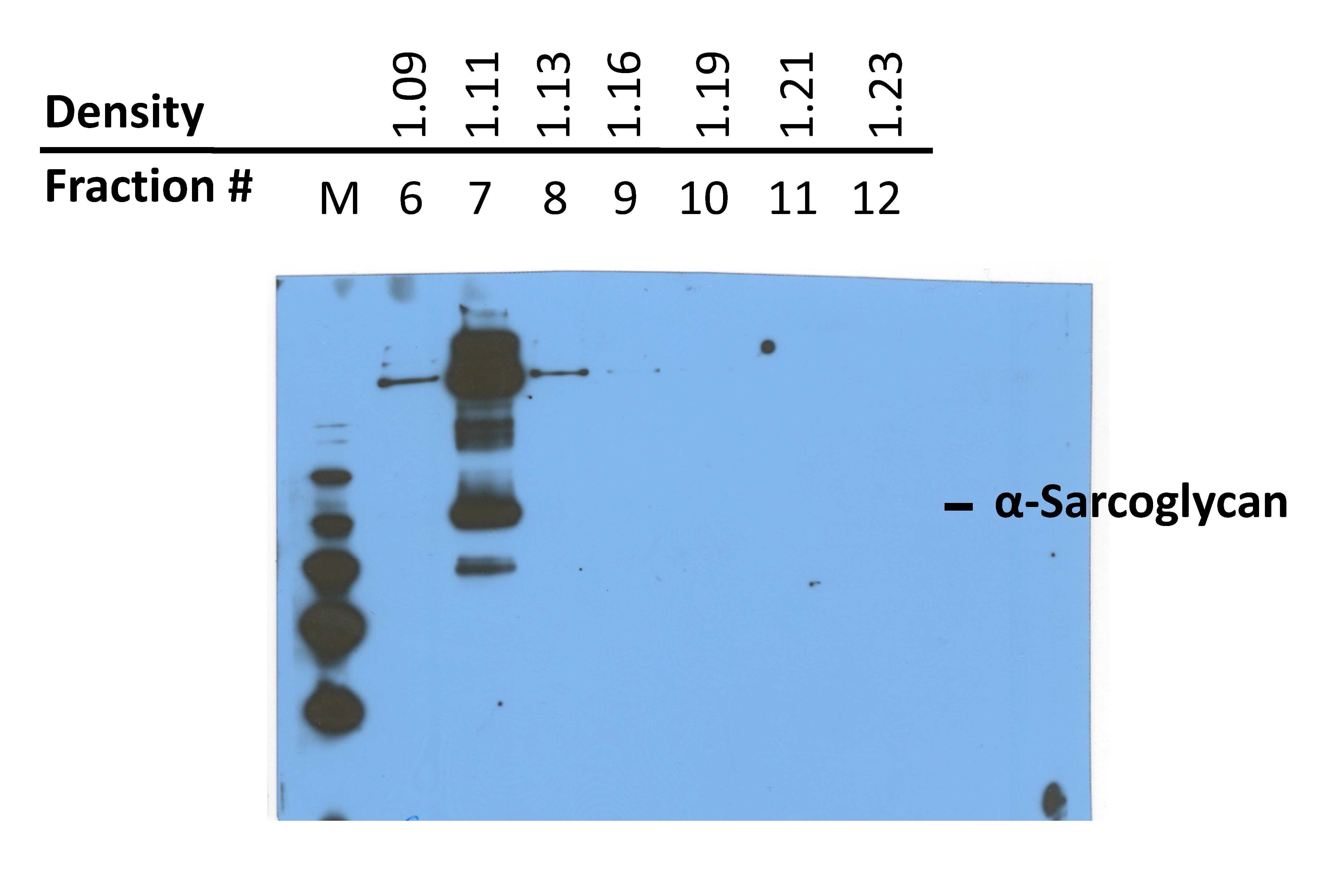

Supplement: S2 Fig — (TIF) [file pone.0125094.s002.tif]

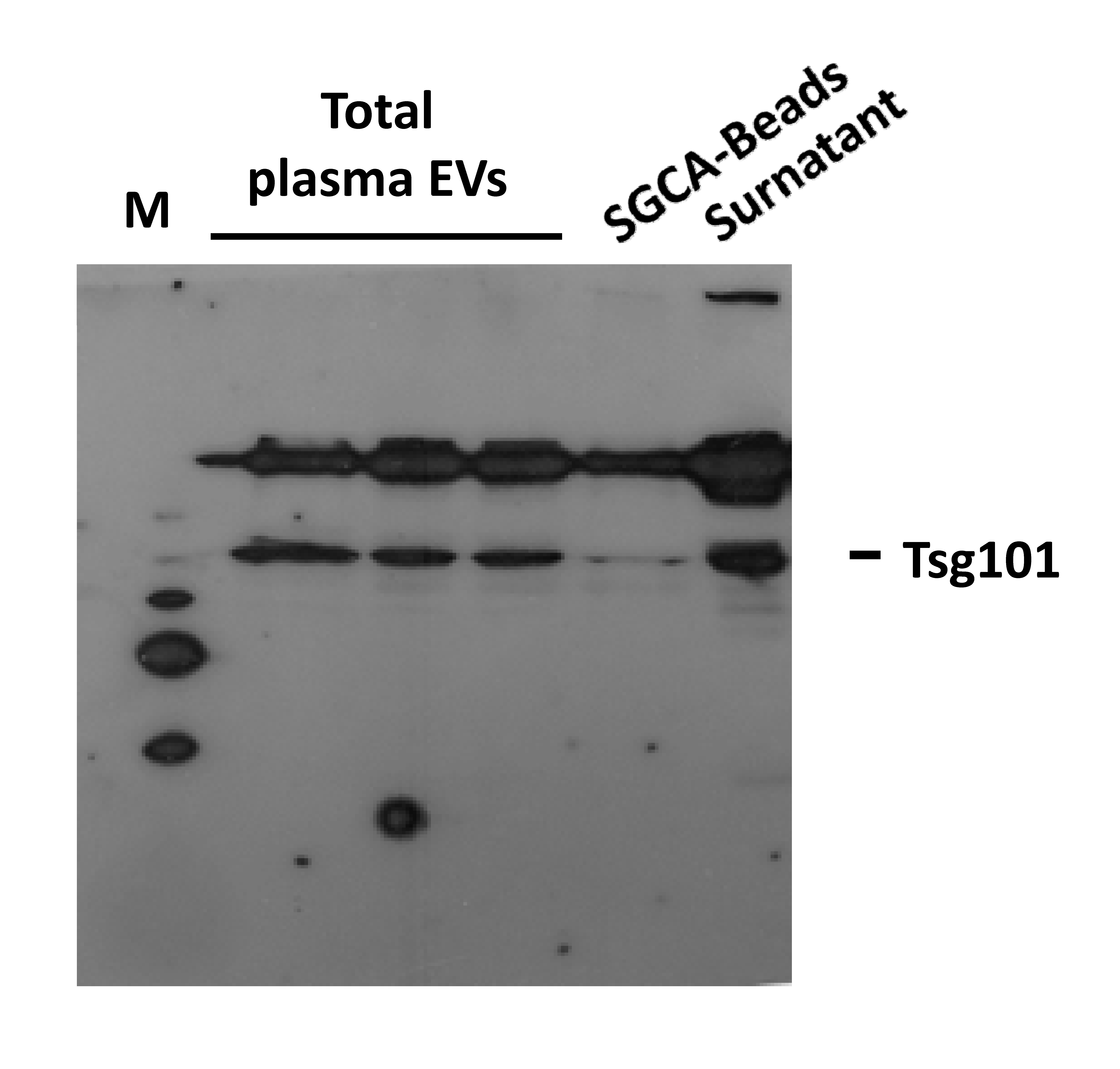

Supplement: S3 Fig — (TIF) [file pone.0125094.s003.tif]
